# A novel microRNA signature predicts survival in liver hepatocellular carcinoma after hepatectomy

**DOI:** 10.1038/s41598-018-26374-9

**Published:** 2018-05-21

**Authors:** Qiang Fu, Fan Yang, Tengxiao Xiang, Guoli Huai, Xingxing Yang, Liang Wei, Hongji Yang, Shaoping Deng

**Affiliations:** 1 Organ Transplantation Center, Sichuan Academy of Medical Sciences and Sichuan Provincial People’s Hospital, School of Medicine, University of Electronic Science and Technology of China, Chengdu, 610072 Sichuan China; 2 Organ Transplantation Translational Medicine Key Laboratory of Sichuan Province, Chengdu, 610072 Sichuan China; 3 Women and Children Health Care Center of Luoyang, Luoyang, 471000 Henan China; 4 People’s Hospital of Changshou Chongqing, Chongqing, 401220 China; 5 Human Islet Laboratory, Massachusetts General Hospital, Harvard Medical School, Boston, 02114 MA USA

## Abstract

Liver hepatocellular carcinoma (LIHC) is the most common type of primary liver cancer. In the current study, genome-wide miRNA-Seq and mRNA profiles in 318 LIHC patients derived from The Cancer Genome Atlas (TCGA) were analysed to identify miRNA-based signatures for LIHC prognosis with survival analysis and a semi-supervised principal components (SPC) method. A seven-miRNA signature was confirmed for overall survival (OS) prediction by comparing miRNA profiles in paired primary tumour and solid tumour normal tissues. Thereafter, a linear prognostic model that consisted of seven miRNAs was established and used to divide patients into high- and low-risk groups according to prognostic scores. Subsequent Kaplan-Meier analysis revealed that the seven-miRNA signature correlated with a good predictive clinical outcome for 5-year survival in LIHC patients. Additionally, this miRNA-based prognostic model could also be used for OS prognosis of LIHC patients in early stages, which could guide the future therapy of those patients and promote the OS rate. Moreover, the seven-miRNA signature was an independent prognostic factor. In conclusion, this signature may serve as a prognostic biomarker and guide LIHC therapy, and it could even be used as an LIHC therapeutic target in the future.

## Introduction

Liver hepatocellular carcinoma (LIHC), as the most common type of primary liver cancer, is the leading cause of the death in adults with cirrhosis^1^. Hepatocarcinogenesis is a complex, multi-step process in which many signalling cascades are altered, leading to a heterogeneous molecular profile and ultimately tumour initiation, progression and metastasis^2^. Even though some effective options, i.e., surgical resection, liver transplantation and ablation, can temporarily improve patients’ quality life quality regardless of the evolutionary stage of their LIHC, overall survival (OS) rate is still poor^3,4^. Tumour node metastasis (TNM) stage and pathological grade are widely used for prognosis, yet challenges remain in accurately staging and grading many tumours, and sub-staging remains controversial^5^. Therefore, it is extremely necessary to identify specific prognostic markers to guide LIHC therapy and improve the OS of LIHC patients.

Accumulating evidence has revealed that microRNAs (miRNAs) play important roles in the development and progression of cancers, such as targeting oncogenes or cancer suppressor genes^6–8^. Possessing specific stability and expression characteristics, miRNAs have been considered useful as biomarkers for cancer prognosis and as potential targets for cancer therapy^9,10^. It has been reported that miRNAs can be used as a prediction model for lymph node metastasis and the recurrence of LIHC after liver transplantation and that miRNA-506 can regulate proliferation, migration and invasion in LIHC by targeting F-spondin 1^11–13^. To date, even though some studies on miRNA-based biomarkers for OS in LIHC patients have been reported, their non-critical criteria may lead to false results^14^.

With the advent of high-throughput sequencing technologies, miRNA profiling datasets have been emerging rapidly. The Cancer Genome Atlas (TCGA) has generated comprehensive, multi-dimensional maps of the key genomic changes in many types of cancer and has created a genomic data analysis pipeline that can effectively collect, select, and analyse human tissues for genomic alterations on a large scale (http://cancergenome.nih.gov/). In the current study, to identify miRNA-based signatures for LIHC prognosis, we analysed the genome-wide miRNA-Seq and mRNA profiles derived from TCGA with bioinformatics methods. First, differentially expressed miRNAs were confirmed by comparing miRNA profiles in paired primary tumour and solid tumour normal tissues to minimize the variations among different individuals^11^. Thereafter, a linear prognostic model consisting of seven miRNAs was established and used to divide patients into high- and low-risk groups according to prognostic scores. Subsequent Kaplan-Meier analysis revealed that the seven-miRNA signature correlated with a good predictive clinical outcome for 5-year survival in LIHC patients, indicating that this signature may guide LIHC therapy and that it could even be used as a therapeutic target in the future.

## Materials and Methods

### TCGA dataset

A total of 1047 miRNA expression profiles in level 3 patients and 24991 mRNAs in LIHC patients along with their corresponding clinical data were downloaded from the TCGA data portal (February 2015). Patients with follow-up longer than 30 days were selected for further analysis to exclude unrelated causes of death.

### Identification of dysregulated miRNAs in LIHC

The raw counts of miRNA expression data of 49 LIHC samples with their paired normal tissues were obtained from the TCGA dataset (Illumina HiSeq Systems). miRNAs whose expression was = 0 in more than 50% of the samples data were removed and were then normalized by log2(X + 1). miRNAs with log2 fold change (log FC) < −1 or log FC > 1 (FDR adjusted *P* < 0.05) were considered to be differentially expressed miRNAs and were included for subsequent analysis.

### Identification of miRNAs with prognostic score in LIHC

To identify common miRNAs related to OS within each of the subgroups stratified by the TMN stage, a semi-supervised method and univariate Cox regression analysis were used, and HR > 1 or HR < 1 with *P* < 0.05 was used as the cutoff^11,15^. The patient subclasses in each group of clinical characteristics represented non-overlapping sets.

### Definition of prognostic risk model and ROC curve analysis

The TCGA data were randomly divided into a training and testing set. A semi-supervised principal components (SPC) method was used to select significant miRNAs and develop the linear miRNA signature prognostic model^11^. The prognostic score was calculated as follows: Prognostic score = (2.307831 × hsa-mir-187) + (hsa-mir-9-3 × 2.553812) + (hsa-mir-490 × 2.726262) + (hsa-mir-1258 × 2.217082) − (hsa-mir-3144 × 2.660158) + (hsa-mir-551a × 3.428998) + (hsa-mir-665 × 2.855964). The prognostic scores were then computed for all 318 patients. The optimal cutoff point of the prognostic score was obtained in ROC curve analysis for predicting the 5-year survival of the training set. Kaplan-Meier and log-rank methods were used for evaluating OS^16^. Time-dependent ROC curves were used to evaluate the predicted power of the miRNA-based signature model.

### Bioinformatic analysis of miRNA-target genes and pathways

The predicted target genes of the candidate miRNAs that existed at least in 4 datasets were obtained from the miRecords v4.0 database (www.mirecords.biolead.org). Differentially expressed miRNAs and differentially expressed genes (DEGs) function and pathway enrichment were analysed using multiple online databases, including DAVID database (https://david.ncifcrf.gov/), which is a multifunctional website for gene annotation, visualization and integrated discovery and can therefore provide gene biological meaning for high-throughput gene functional analysis^17^. Kyoto Encyclopedia of Genes and Genomes (KEGG, http://www.genome.jp/) pathways are a computational representation of the biological system, including gene functions linked to genomic information with higher-order functional information^18^. KEGG pathway enrichment analysis was conducted with Cytoscape (version: 3.6.0). The results were considered statistically significant when the p-value < 0.05 after the FDR was corrected.

## Results

### Identification of differentially expressed miRNAs in LIHC

After filtering out unqualified cases with a follow-up of less than 30 days, a total of 318 LIHC patients (213 male/105 female) were retained for survival analysis, among which was a total of 49 patients with solid tumour normal tissues. These cases were randomly divided into a training set (n = 159) and a testing set (n = 159). There was no significant difference in clinical covariates observed between the two sets (Table 1). Differentially expressed analysis in 49 pairs of primary tumour and solid tumour normal tissues indicated that 156 miRNAs were differentially expressed (log FC > 1 or log FC < −1, *P* < 0.05 after FDR adjustment). Among these miRNAs, 37 miRNAs were up-regulated, and 119 miRNAs were down-regulated.

**Table 1:** Clinical covariates for the TCGA LIHC cohort in the training and testing set.

Covariates		Total	Training set	Testing set	P-value
		N = 318	N = 159	N = 159	
Age (years), no (%)	<65	177 (55.7)	86 (54.1)	91 (57.2)	0.6517
	≥65	141 (44.3)	73 (45.9)	68 (42.8)	
Sex, no (%)	Male	213 (66.8)	110 (69.2)	103 (64.8)	0.4039
	Female	105 (33.2)	49 (30.8)	56 (35.2)	
Vital status, no (%)	Alive	209 (65.5)	106 (66.7)	103 (64.8)	0.723
	Dead	109 (34.5)	53 (33.3)	56 (35.2)	
Stage, no (%)	I	161 (50.5)	82 (51.6)	79 (49.7)	0.8894
	II	74 (23.2)	38 (23.9)	36 (22.6)	
	III	58 (18.5)	26 (16.4)	32 (20.1)	
	IV	5 (1.6)	2 (1.3)	3 (1.9)	
	Unknowm	20 (6.3)	11 (6.9)	9 (5.7)	
T stage, no (%)	T0	1	1 (0.6)	0 (0.0)	0.6515
	T1	169 (53.0)	85 (53.5)	84 (52.8)	
	T2	80 (25.1)	38 (23.9)	42 (26.4)	
	T3	55 (17.6)	28 (17.6)	27 (17.0)	
	T4	11 (3.4)	5 (3.1)	6 (3.8)	
	Tx	2 (0.9)	2 (1.3)	0 (0.0)	
N stage, no (%)	N0	222 (69.9)	110 (69.2)	112 (70.4)	0.1933
	N1	3 (0.9)	0 (0.0)	3 (1.9)	
	Nx	93 (29.2)	49 (30.8)	44 (29.6)	
M stage, no (%)	M0	229 (72.1)	114 (71.7)	115 (72.3)	0.8446
	M1	3 (0.9)	2 (1.3)	1 (0.6)	
	Mx	86 (27.0)	43 (27.0)	43 (27.7)	
Adjuvant treatment, no (%)	Ablation embolization + Pharmaceutical therapy	5 (1.6)	3 (1.9)	2 (1.3)	0.3566
	Pharmaceuticaltherapy	19 (6.3)	6 (3.8)	13 (8.2)	
	Radiation + Pharmaceutical therapy	1 (0.3)	0 (0.0)	1 (0.6)	
	Ablation embolization	17 (6.6)	11 (6.9)	6 (3.8)	
	Radiationtherapy	7 (2.2)	2 (1.3)	5 (3.1)	
	None	269 (83.1)	137 (86.2)	132 (86.2)	

### Establishment of the miRNA prognostic model

**Figure 1:**
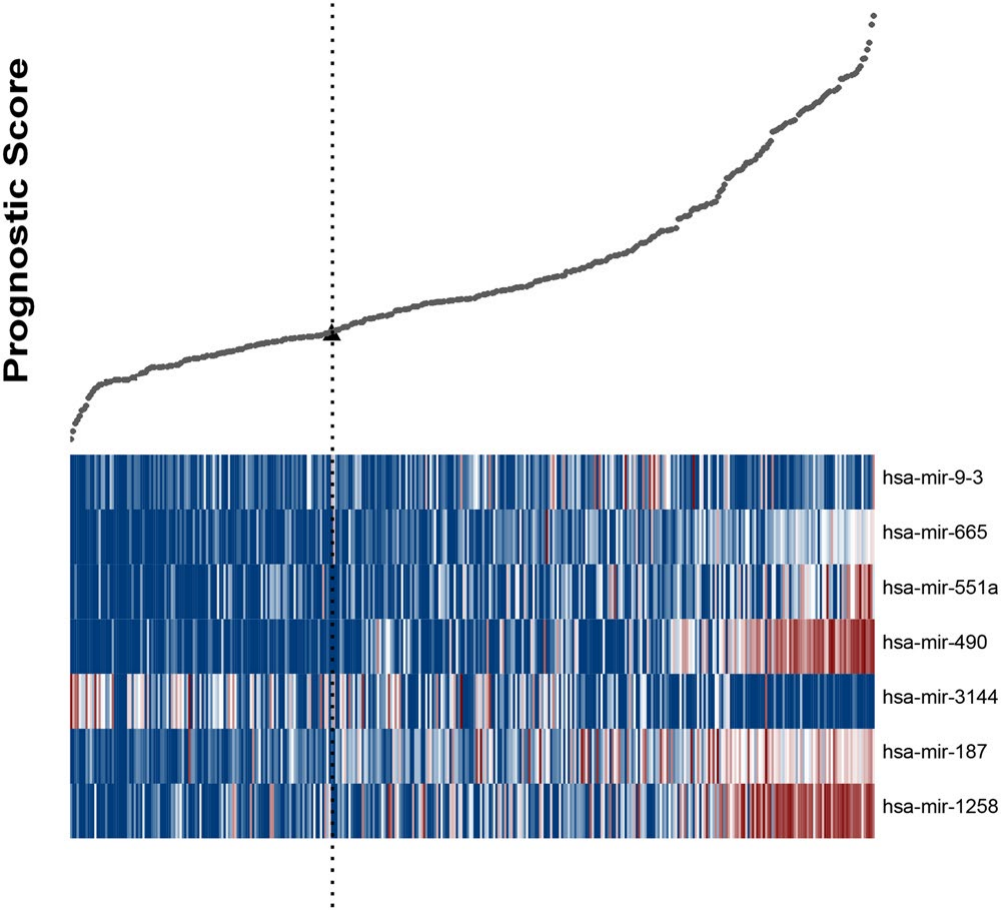
Heatmap and predictor-score of seven-miRNA signature of LIHC cohort. (**A**) MicroRNA predictor-score distribution. (**B**) Heatmap of seven-miRNAs expression profiles of LIHC patients.

The common miRNAs associated with OS and tumourigenesis were characterized with the univariate Cox regression method^16^. Seven miRNAs were selected using the supervised principal component method in the training set. Thereafter, we developed a miRNA prognostic model for diagnosis in the LIHC cohort. According to ROC curve, the optimum cutoff point was calculated and used for classifying the samples into high-risk and low-risk groups (Fig. 1). This analysis showed the prognostic scores and miRNA expression distribution of all 318 patients and 49 pairs of solid tumour normal tissues, which were ranked according to the prognostic scores for the seven-miRNA signature. It indicated that the patients with higher prognostic scores showed a tendency towards expression of high-risk miRNAs, whereas patients with low prognostic scores showed a tendency towards non-protective miRNA expression.

### Validation of the seven-miRNA signature in LIHC patients

**Figure 2:**
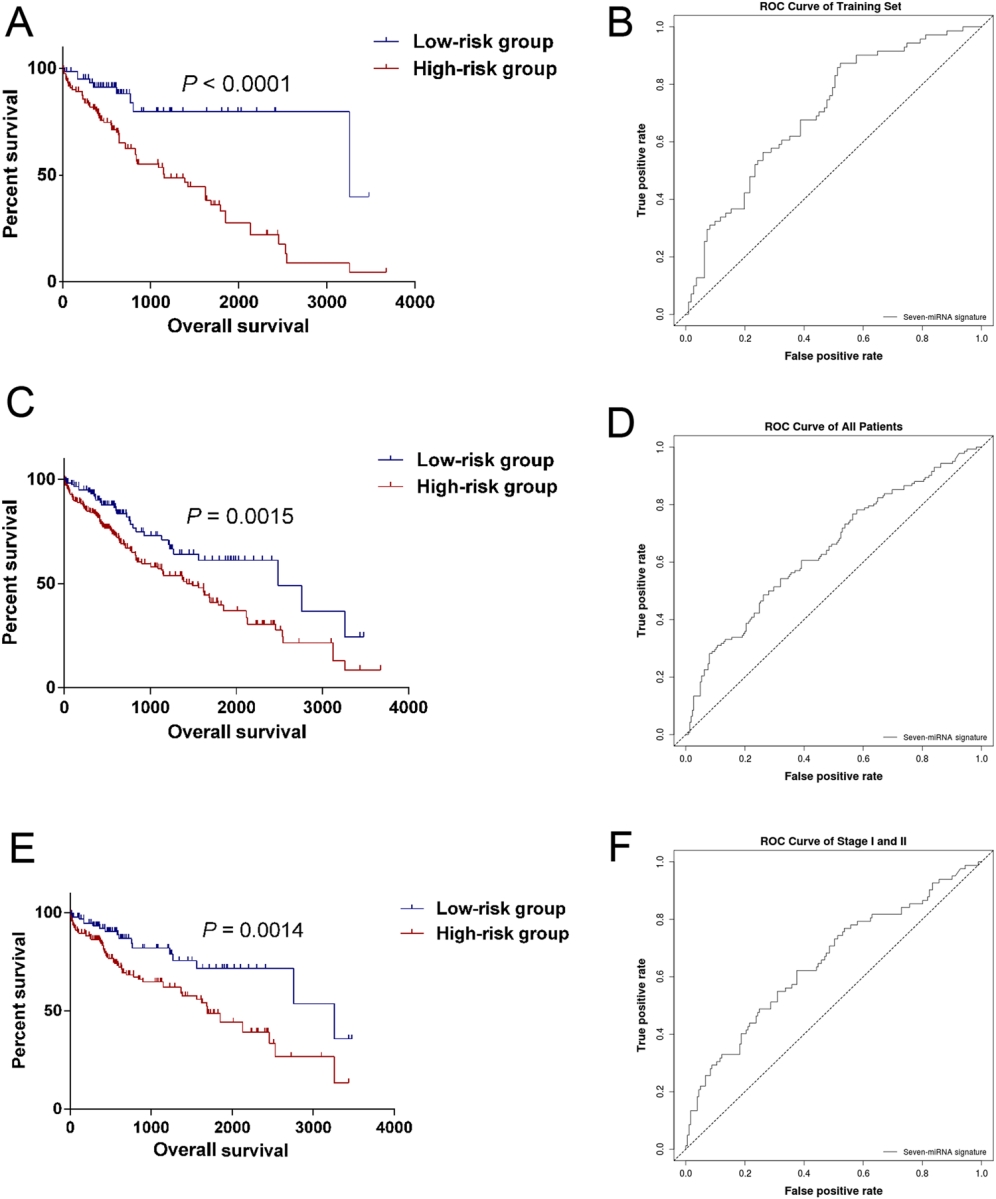
Kaplan–Meier and ROC curves for the seven-miRNA signature in LIHC patients. The Kaplan–Meier curves for training set (**A**), entire LIHC cohort (**C**) and in early stages (**E**) divided by the optimum cutoff point. Patients with high scores had the poor outcome in terms of OS (Median OS of testing set: 3258 days vs. 1153 days, *P* < 0.0001; Median OS of entire LIHC cohort: 2486 days vs. 1490 days, *P* = 0.0015; Median OS of entire LIHC cohort: 3258 days vs. 1294 days, *P* = 0.0014). The ROC curve for predicting 60-month survival for training set (**B**), entire LIHC cohort (**D**) and patients in early stages.

Using the optimum cutoff value obtained from the training set, patients were assigned to high-risk and low-risk groups. The ability of the seven-miRNA signature to predict 5-year survival prognostication was examined in the testing set and in all LIHC patients. The results indicated that patients in the high-risk group had poor OS, whereas patients in the low-risk group had good outcomes in the training set (*P* < 0.001, with the log-rank test method, Fig. 2A) and in the entire LIHC cohort (*P* = 0.0015, Fig. 2C). The AUC of the seven-miRNA signature prognostic model of the time-dependent ROC curve in the training set was 0.703 (Fig. 2B), whereas the AUC for all LIHC patients was 0.645 (Fig. 2D). To further confirm the accuracy of the prognostic model, AUC was calculated with Kaplan-Meier methods in the early stage (including stage I and II) patients (n = 263). This calculation showed a similar result to that of the training set and the entire LIHC cohort (*P* = 0.0014, Fig. 2E), and the AUC of the ROC curve was 0.652 (Fig. 2F). Therefore, this model could be helpful for predicting the survival of STAD patients in stage I and II.

### The seven-miRNA signature is an independent prognostic factor

To identify whether the seven-miRNA signature is an independent factor that is not affected by other factors, such as age, neoplasm histologic grade or TNM stage, Cox multivariate regression analysis was performed, and the result indicated that the seven-miRNA signature was indeed an independent prognostic factor (Table 2; HR = 1.034 and *P* = 0.007).

**Table 2:** Multivariate analysis of overall survival of patients.

Characteristic	Hazard ratio	Lower limit (95%)	Upper limit (95%)	p value
Gender (male vs. female)	0.977	0.681	1.404	0.902
Age (<65 vs. ≥65 years)	1.219	0.861	1.726	0.265
Grade (Grade 1–2 vs 3–4)	1.64	1.125	2.391	**0.01**
T stage (T1–2 vs T3–4)	1.953	1.361	2.802	0
N stage (N0 vs N1–3)	1.164	0.718	1.886	0.539
M stage (M0 vs M1)	2.099	1.307	3.371	**0.002**
miRNA signature	1.034	1.009	1.06	**0.007**

### In silico analysis of target genes, pathways and DEGs

**Figure 3:**
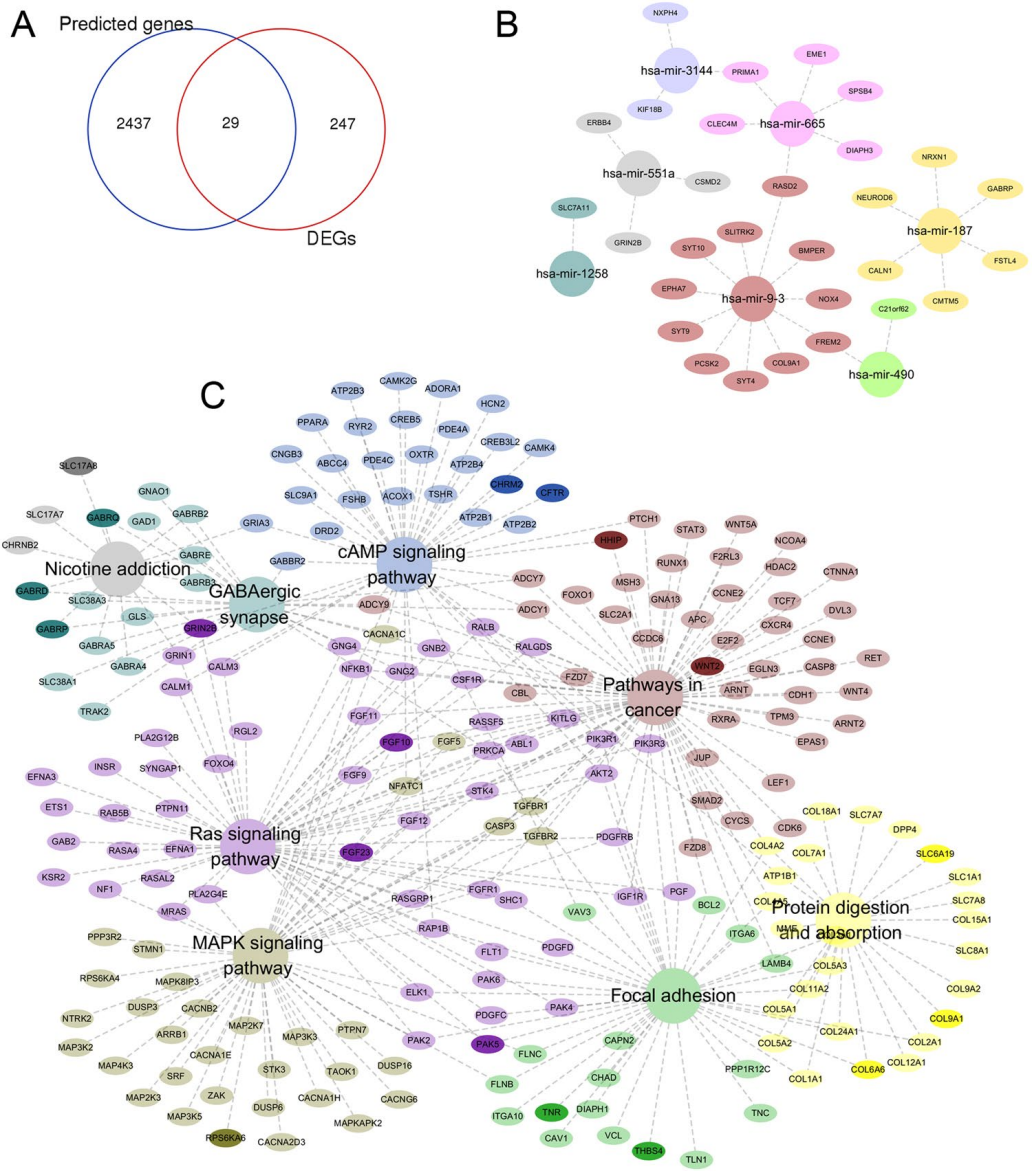
In silico analysis of target genes, pathways. (**A**) There were 29 DEGs were predicted by the seven-miRNA signature. (**B**) Those 29 DEGs were respectively predicted and regulated by the seven miRNAs. (**C**) KEGG enrichment analysis results showed that those genes mainly were involved in 8 KEGG pathways (*P* < 0.05 after FDR adjustment), and deep color represented 18 DEGs, which were enriched and involved in those 8 KEGG pathways.

**Figure 4:**
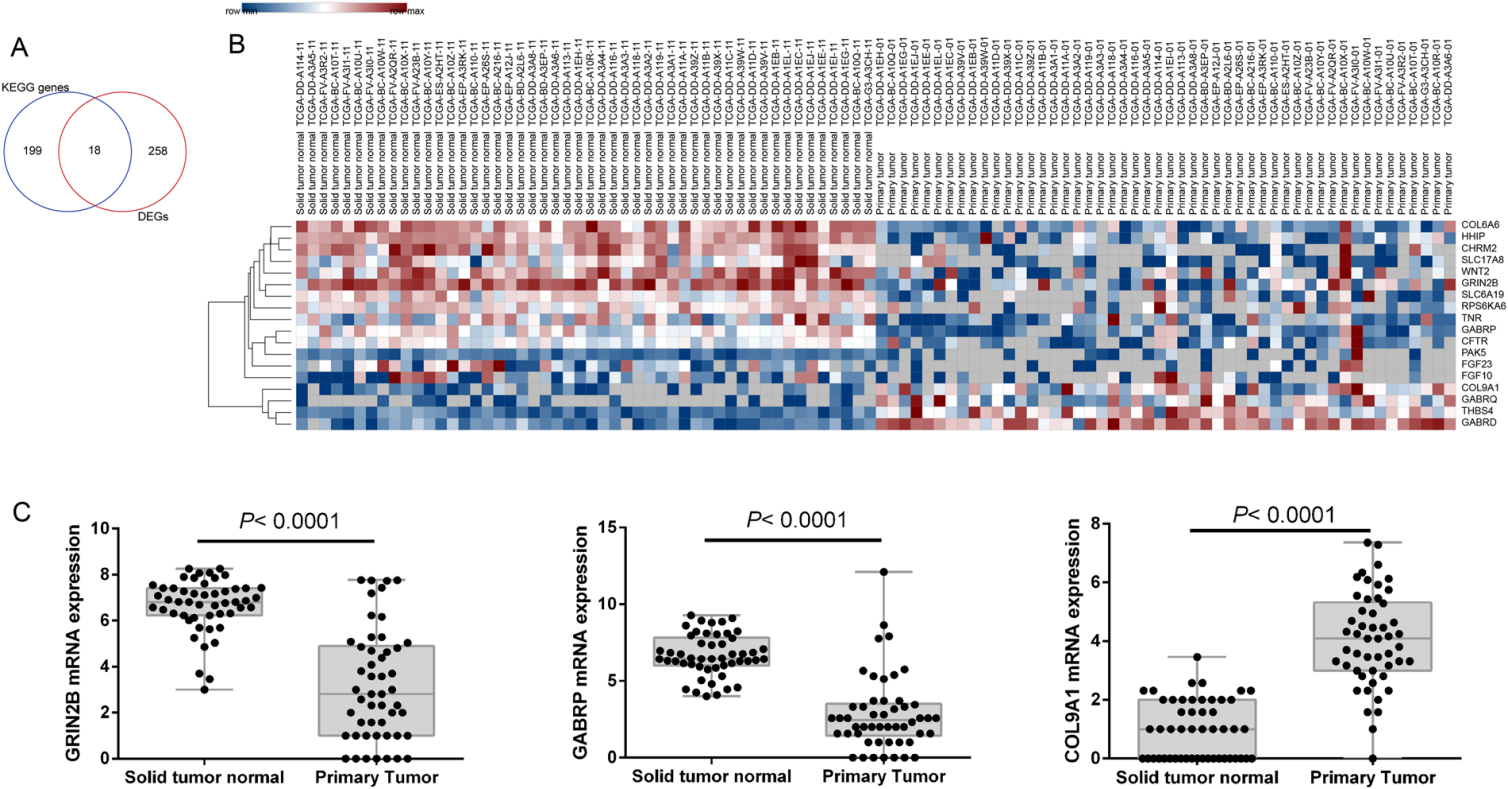
Results of pathway analysis of the target genes. (**A**) In the enriched KEGG genes, 18 ones were confirmed as DEGs. (**B**) Heat map of the miRNA expressions of all 18 DEGs. (**C**) MiRNA expression of miR-187 targeted GABRP, miR-551a targeted GRIN2B and miR-9-3 targeted COL9A1.

The targeted genes, which might be potentially regulated by the seven miRNAs and were predicted by more than 4 datasets were downloaded from miRecords and analysed. In addition, 276 differentially expressed genes (DEGs) were found. Twenty-nine of these DEGs were also found in the 2466 predicted genes (Fig. 3A) that were regulated by the seven miRNAs (Fig. 3B). KEGG enrichment results demonstrated that these genes were mainly involved in 8 KEGG pathways, which included the Ras signalling pathway, protein digestion and absorption, pathways in cancer, cAMP signalling pathway, focal adhesion, MAPK signalling pathway, GABAergic synapse and nicotine addiction (*P* < 0.05 after FDR adjustment, Fig. 3C). In these enriched pathways, a total of 18 genes were changed significantly (log FC > 1 or <−1, and *P* < 0.05 after FDR adjustment, Fig. 4A,B), proving the relevant pathways and mechanisms indirectly. Specifically, three genes, including GABRP, GRIN2B and COL9A1, were predicted by miR-187, miR-551a and miR-9-3, respectively (Fig. 4C).

## Discussion

When LIHC occurs, it always accompanies chronic liver disease, such as cirrhosis. It may result in a worsening of symptoms, or it sometimes presents without symptoms at cancer detection^19^. Therefore, understanding the molecular mechanisms of LIHC is critical for LIHC diagnosis, prevention and therapy. In our study, 156 differentially expressed miRNAs were found between LIHC primary tumour and solid tumour normal tissues. A seven-miRNA signature (miR-187, miR-9-3, miR-490, miR-1258, miR-3144, miR-551a and miR-665) was identified and confirmed as a predictor of OS in the LIHC patients, especially patients in early stages (stage I and II), using survival analysis, ROC curve and SPC methods. Additionally, it was further confirmed that the seven-miRNA signature was an independent prognostic factor. Among these seven miRNAs, six were negatively associated with survival and one was positively associated.

Six negative-relevant miRNAs were miR-187, miR-9-3, miR-490, miR-1258, miR-551a and miR-665; among these miRNAs, miR-187 has been found to be overexpressed in ovarian cancers^20^, can induce epithelial-mesenchymal transition (EMT) by targeting PTRF in non-small cell lung cancer^21^ and promotes the growth and metastasis of gastric cancer by inhibiting FOXA2^22^. this result indicated that miR-187 possessed the potential to be a candidate for LIHC therapy. miR-9-3 was also confirmed to be a risk factor that can lead to poor 5-year survival. A previous report revealed that the upregulation of miR-9 expression can predicate advanced clinicopathological features and poor prognosis in LIHC patients, which was in accordance with the current results^23^. However, it has been reported that miR-9-3 acts as a tumour suppressor by targeting TAZ in LIHC^9^. This discrepancy among these studies may be caused by subtle variations in the patients, such as differences in race and living conditions. miR-490, another risk factor, has been reported to suppress tumourigenesis and progression in endometrial carcinoma by targeting TGFα^24^ and to modulate cell growth and EMT of LIHC cells by targeting ERGIC3. We found that miR-490 was up-regulated, which meant that inhibition of this oncogenic miRNA might provide a potential approach for LIHC therapy. miR-1258 and miR-551a were two other risk factors identified in this study. However, it has been proven that miR-1258 is significantly down-regulated in breast cancer^25^ and that miR-551a can suppress cell migration and invasion in gastric cancer by targeting PRL-3^26^, which is inconsistent with our current result, and this inconsistency might be due to different cancer types. It has been reported that miR-665 plays a key role in inflammatory bowel disease and tumour metastatic dissemination^27,28^, which means that miR-665 might be used as a potential target in LIHC therapy. As the only protective miRNA in the current study, miR-3144 can suppress target reporter activity markedly and can inhibit the expression of both synthetically exogenous and endogenous E6 oncogenes and ultimately inhibit cell growth and promote apoptosis in human papillomavirus 16 (HPV16)-positive cervical cancer cells^29^. As a promising common target of miRNAs for most high-risk HPVs, lowly expressed miR-3144 might play a key role in LIHC suppression. However, further work is still needed to validate its prognostic and target roles for clinical applications.

It is worth noting that both ROC curve and survival analyses of the early stage (stage I and II) LIHC cases proved that the seven-miRNA signature could predict their high risk and 5-year OS in early stages, which accounts for almost 60% (74/125) of all recurrent LIHC patients. Therefore, it may be beneficial for these cases to use this prognostic model to guide further medical interventions, as its results can potentially shorten exam intervals for recurrence monitoring and be used to adjust other therapy plans promptly. In silico analysis of target genes, KEGG pathways and DEGs showed that several meaningful pathways were involved in LIHC development and progression, such as the Ras signalling pathway. In all 2466 predicted genes, 29 genes were identified as DEGs in the current study. In all 217 enriched genes, 18 genes were proven to be differentially expressed. Three genes, including GABRP, GRIN2B and COL9A1, were predicted by miR-187, miR551a and miR-9-3, respectively. Specifically, GRIN2B was enriched in three pathways, including the Ras signalling pathway, cAMP signalling pathway, and nicotine addiction. While it was first identified in oesophageal squamous cell carcinoma^30^, GRIN2B has been subsequently confirmed in many other cancer types, such as oesophageal cancer, gastric cancer and breast cancer^31–33^. GRIN2B is epigenetically inactivated and supresses oesophageal cancer activity^33^. However, it has been reported that active GRIN2B has an important influence on the survival of breast cancer patients and that GRIN2B methylation is a common and important biologically relevant event in gastric cancer progression^31,32^. In the current study, GRIN2B was down-regulated significantly, which meant that miR-551a overexpression could supress GRIN2B activity and worsen the LIHC patient’s condition. Antagonists targeting MiR-551a or a GRIN2B activator might be used as potential candidates in LIHC therapy in the future. GABRP, which was mainly enriched in GABAergic synapses and nicotine addiction, was progressively down-regulated with tumour-progression in breast cancer, which meant that it might be used as a prognostic marker^34^. Interestingly, it has also been reported that GABRP can stimulate basal-like breast cancer cell migration through the activation of ERK1/2^35^. The completely opposite results may be due to sample numbers or patients of different races. In our study, miR-187-targeted GABRP was significantly down-regulated and could be used as a prognostic marker for LIHC. To date, there have not been any reports about COL9A1 function in the progression of LIHC. We found that COL9A1 was mainly enriched in focal adhesions, which play an important role in LIHC^36,37^. In this study, miR-9-3-targeted COL9A1 was significantly up-regulated and could be useful as a prognostic marker for LIHC survival. All data were indirect proof of the usefulness of the seven-miRNA signature.

In summary, a specific seven-miRNA signature prognostic model was identified using the TCGA dataset, and our validation cohort confirmed that the prognostic model may be a useful biomarker in LIHC patients. However, prospective, large-scale, multicentre studies are still needed for further validation before the current results can be applied in clinical settings.
