# Author Correction: A novel microRNA signature predicts survival in liver hepatocellular carcinoma after hepatectomy

**DOI:** 10.1038/s41598-018-27641-5

**Published:** 2018-06-15

**Authors:** Qiang Fu, Fan Yang, Tengxiao Xiang, Guoli Huai, Xingxing Yang, Liang Wei, Hongji Yang, Shaoping Deng

**Affiliations:** 1 Organ Transplantation Center, Sichuan Academy of Medical Sciences and Sichuan Provincial People’s Hospital, School of Medicine, University of Electronic Science and Technology of China, Chengdu, 610072 Sichuan China; 2 Organ Transplantation Translational Medicine Key Laboratory of Sichuan Province, Chengdu, 610072 Sichuan China; 3 Women and Children Health Care Center of Luoyang, Luoyang, 471000 Henan China; 4 People’s Hospital of Changshou Chongqing, Chongqing, 401220 China; 5 Human Islet Laboratory, Massachusetts General Hospital, Harvard Medical School, Boston, 02114 MA USA

Correction to: *Scientific Reports* 10.1038/s41598-018-26374-9, published online 21 May 2018

The Acknowledgements section in this Article is incomplete.

“We thank the TCGA project, which generated comprehensive, multi-dimensional maps of key genomic changes in liver hepatocellular carcinoma. This work was supported by grants from the Science and Technology Department of Sichuan Province, No. 30504010361 and No. 2014FZ0123 and grants from the National Natural Science Foundation of China, No. 81571565.”

should read:

“We thank the TCGA project, which generated comprehensive, multi-dimensional maps of key genomic changes in liver hepatocellular carcinoma. This work was supported by grants from the Science and Technology Department of Sichuan Province, No. 30504010361 and No. 2014FZ0123, grants from the National Natural Science Foundation of China, No. 81571565 and grants from the Health and Family Planning Commission of Sichuan Province, No. 2015SZ0241.”

